# Hypertension and the development of New onset chronic kidney disease over a 10 year period: a retrospective cohort study in a primary care setting in Malaysia

**DOI:** 10.1186/1471-2369-13-173

**Published:** 2012-12-24

**Authors:** Yook Chin Chia, Siew Mooi Ching

**Affiliations:** 1 Department of Primary Care Medicine, Faculty of Medicine, University of Malaya, Kuala Lumpur 50603, Malaysia; 2 Curtin Health Innovation Research Institute, Faculty of Health Sciences, University of Curtin, GPO Box U1987, Perth, Western Australia 6845, Australia; 3 Department of Family Medicine,Faculty of Medicine and Health Sciences, Universiti Putra Malaysia, Serdang, Selangor, 43400, Malaysia

**Keywords:** Hypertension, Chronic kidney disease, Primary care, Estimated glomerular filtration rate, Cohort, Malaysia

## Abstract

**Background:**

Little is known about the rate of progression to chronic kidney disease (CKD) among hypertensive patients, particularly at the primary care level. This study aims to examine risk factors associated with new onset CKD among hypertensive patients attending a primary care clinic.

**Methods:**

This is a 10-year retrospective cohort study of 460 patients with hypertension who were on treatment. Patient information was collected from patient records. CKD was defined as a glomerular filtration rate <60 ml/min per 1.73 m^2^ (Cockcroft-Gault equation). Multiple logistic regression statistics was used to test the association in newly diagnosed CKD.

**Results:**

The incidence of new CKD was 30.9% (n = 142) with an annual rate of 3%. In multivariate logistic regression analysis, factors associated with development of new onset of CKD among hypertensive patients were older age (odds ratio [OR] 1.123, 95% confidence interval [CI] 1.078-1.169), presence of diabetes (OR 2.621, 95% CI 1.490-4.608), lower baseline eGFR (OR 1.041, 95% CI 0.943-0.979) and baseline hyperuricaemia (OR 1.004, 95% CI 1.001-1.007).

**Conclusions:**

The progression to new onset CKD is high among urban multiethnic hypertensive patients in a primary care population. Hence every effort is needed to detect the presence of new onset CKD earlier. Hypertensive patients who are older, with underlying diabetes, hyperuricaemia and lower baseline eGFR are associated with the development of CKD in this population.

## Background

Chronic kidney disease (CKD) is recognised as one of the global public health problems, particularly in Asia with its looming epidemic of diabetes mellitus and rapid increase in the ageing population [1-3]. Hypertension has been shown to be a risk factor for CKD worldwide [4-6] and CKD is recognised as one of the independent risk factors for cardiovascular disease (CVD) [7,8]. Several studies have shown that those who have reduced renal function, as determined by a reduction in glomerular filtration or presence of albuminuria, are at a higher risk of CVD mortality and morbidity [9-12]. In hypertension, glomerular filtration has been reported to decline more rapidly at a rate of 1.5 mL/ min per 1.73 m^2^ every year compared to those without hypertension whose decline is at 0.75–1.00 mL/ min per 1.73 m^2^ every year after the age of 40 years [13-15]. Although previous studies have shown that only a small percentage where 15.6 cases per 100,000 person-years of hypertensive patients develop end-stage renal failure [16,17], it is still one of the most important factors associated with the progression of both diabetic and nondiabetic CKD [18]. Hence, it is important for clinicians to be able to identify patients who are at high risk of developing CKD for intensification of treatment. This will not only help to prevent or delay further deterioration of renal function but also to reduce the cardiovascular risk [5,7,8,19].

However, there are very few studies that look at the decline rate and the development of new onset CKD over time in patients with hypertension. Thus, the aim of this study was to determine the rate of change of glomerular filtration over time and the development of new onset CKD over a 10-year period in patients with hypertension attending a primary care clinic.

## Methods

### Setting

The current research is part of a 10-year retrospective cohort study of patients registered with the Department of Primary Care Medicine Clinic at the University of Malaya Medical Centre (UMMC). Ethics approval was obtained from the Ethics Committee of University of Malaya Medical Centre.The cohort was randomly selected from the clinic patient records based on numbers generated by a computer programme. Baseline data was collected in 1998, and follow-up data collected in 2002 and 2007 at the five-year intervals.

### Inclusion criteria

Out of the original cohort, adults aged 30 years or older with hypertension and with eGFR ≥60 ml/min/1.73 m^2^ in 1998 were identified and selected for this study. Patients with eGFR < 60 ml/min/1.73 m^2^ and diabetes without hypertension were excluded.

The estimated glomerular filtration rate (eGFR) was used to determine renal function [20] and it was based on the Cockcroft-Gault formula as follows [21].

$$\mathrm{eGFR}\left(\mathrm{ml}/\min\right)=\frac{\left[\left(140-\mathrm{age}\right)\times \mathrm{weight}\left(\mathrm{kg}\right)\right]}{\left(\mathrm{Serum}\;\mathrm{Creatinine}\left(\mathrm{umol}/L\right)/88.4\right)\times 72}\times 0.85\;\mathrm{if}\;\mathrm{female}$$

### Data collection

Socio-demographic data and comorbidities were also recorded. Weight and blood pressure (BP) were captured. BP was measured by mercury sphymomanometer. Hypertension was defined as those who had a documented diagnosis of hypertension (i.e. BP ≥ 140/90 mmHg) or those on anti-hypertensive agents. Target control BP was defined as BP < 140/90 mmHg for non-diabetic and < 130/80 mmHg for diabetic patients. Body mass index (BMI) was calculated as weight in kilograms per square meter (kg/m^2^).

Diabetes mellitus was defined as self-reported diabetes or the use of hypoglycemic agents or both. Smokers were defined as current if they were still smoking or non smokers as those who never smoked or currently not smoking regardless of when they had stopped smoking. Anti-hypertensive drug use was also captured and classified into the following classes: renin-angiotensin-system (RAS) inhibitors encompassing angiotensin-converting enzyme inhibitors (ACEI), angiotensin receptor blockers (ARB); beta-blockers (ß-blockers), calcium-channel blocker (CCB), diuretics and alpha-blockers (α-blockers). Statin use was recorded as well. Serum creatinine, uric acid and Haemoglobin A1C (HbA1c) levels were also collected.

### Classification of chronic kidney disease (CKD)

We categorised the patients into two groups: Those who deteriorated i.e. eGFR < 60 ml/min per 1.73 m^2^ (stage 3–5) and those who did not deteriorate i.e. eGFR ≥60 ml/min per 1.73 m^2^ (Stage 1 and 2) at the end of the 10 years. The categorization was done in this way because when eGFR is <60mil/min per 1.73 m^2^, half of the function of the kidney is lost and it is at this point when the prevalence of complications are increased [22,23]. The rate of decline in GFR was calculated as the difference in the eGFR between 2007 and 1998 divided by the 10 years of follow-up.

### Statistical analysis

All statistical analysis was done using the Statistical Package for Social Sciences (SPSS version 15). Continuous data are described as mean and standard deviation if the distribution is normal. When the data was a skewed distribution, median and interquartile range (25-75^th^ percentiles) was used. Categorical data are reported as proportions (percentage). Chi-square test or Fisher exact tests were used for the categories or dichotomous predictors. Multivariate logistic regression analysis was used to look for the predictors of new onset of CKD. All analyses were done with 95% confidence intervals (CI), and the level of significance was set at p < 0.05.

## Results

A total 1547 patients were in the original cohort; 886 patients were hypertensive with or without diabetes. After excluding missing data on eGFR in 1998 (n = 62), baseline eGFR < 60 ml/min/1.73 m^2^ at 1998 (n = 211) and missing data on eGFR in 2007(n = 153), finally 460 patients who had complete data and who fulfilled the inclusion criteria were entered into our analysis. We also analysed those with the missing data (n = 153) and found that their baseline characteristics were similar to those patients who had the complete data set. For those 460 patients, 312 (68%) had hypertension alone and a further 148 (32%) had both diabetes and hypertension.

Overall, the baseline mean age of the patients was 54.9 ± 9.0 years (range 30 to 87 years), 43.8% were male and 13.3% were aged ≥65 years. The ethnic distribution was 25.4% Malays, 50.0% Chinese and 23.5% Indians. 6.5% were smokers. At baseline, the mean BP was 145/88 ± 18/10 mmHg and BMI was 27.8 ± 4.4 kg/m^2^ while the mean eGFR was 87 ± 24 mL/min/1.73 m^2^.

Table 1 shows the change in mean BP, control rate of blood pressure, mean eGFR and use of antihypertensive agents over the three-time intervals. There was an improvement of mean BP over the 10 years with more patients reaching target BP at the end of the 10-year period. However, in diabetics, at baseline less than 1% achieved BP control. After 5 years, the control of BP was not much better at 1% and at the end of 10 years only 12.1% achieved BP control. (Table 1) The mean decline rate in glomerular filtration for the whole group was −1.26 ± 2.4 ml/min per year. However, in the group of hypertensive patients with diabetes, the decline rate was two times higher at −1.98 ± 2.51 ml/min per year than those hypertensives without diabetes, where their decline rate of eGFR of −0.92 ± 2.22 ml/min per year was much lower (p-value < 0.001).

**Table 1:** Demographic and Clinical Characteristics of the study populations at baseline, 5 years and 10 years (N = 460)

**Variable**	**1998**	**2002**	**2007**
Mean systolic BP (mmHg)	145 ± 18	142 ± 17	135 ± 16
Mean diastolic BP (mmHg)	88 ± 10	84 ± 9	80 ± 8
Patients with HPT and DM (n, %)	148 (32)	207 (45)	239 (52)
Overall BP control rate (n, %)	70 (15.2)	87 (18.9)	189 (41.1)
BP control rate in HPT with DM (n, %)	1 (0.7)	2 (1.0)	29 (12.1)
Use of RAS blockers (n, %)	52 (11.3)	111 (24.1)	227 (49.3)
Mean number antihypertensive agent (n)	1.2	1.8	2
Mean eGFR (mL/min per 1.73 m^2^)	87 ± 24	76 ± 22	75 ± 28
eGFR stage 3, <60 mL/min per 1.73 m^2^ (n, %)	0	87 (18.9)	142 (30.9)
eGFR stage 4, <30 mL/min per 1.73 m^2^ (n, %)	0	1 (0.2)	8 (1.7)

BP: blood pressure, HPT: Hypertension, DM: Diabetes Mellitus, RAAS: renin-angiotensin aldosterone system, eGFR: estimated glomerular filtration rate.

The number of patients who deteriorated from a baseline eGFR of ≥ 60 ml/min/1.73 m^2^ to eGFR < 60 ml/min/1.73 m^2^ at the end of 10 years was 30.9%. The lowest eGFR seen at the end of 10 years was 15.7 ml/min/1.73 m^2^. However none of the patients developed end stage renal disease (ESRD) and none needed dialysis at any time throughout the 10 years. Among the hypertensive patients with diabetes, there was no difference in the HbA1C level in those who developed CKD and those who did not develop CKD (HbA1C 7.5% ± 1.5 mmol/L and 7.4% ± 1.7 (p = 0.78) respectively).

Table 2 compares the characteristics of those who deteriorated and those who did not. Those who deteriorated were those who at baseline had lower eGFR but higher serum uric acid level, being older, and had underlying diabetes.

**Table 2:** Association of baseline characteristics between patients who deteriorated and those who did not deteriorate (N = 460)

	**No deterioration**	**Deteriorated**	
**Baseline Variables in 1998**	**eGFR ≥ 60 ml/min (n = 318)**	**eGFR < 60 ml/min (n = 142)**	**p-value**
Age, years	52.3 ± 7.9	60.5 ± 7.9	<0.001
Males (n, %)	99 (31.1)	63 (44.7)	0.005
Race, Malay (n, %)	82 (25.9)	34 (24.1)	0.889
Race, Chinese (n, %)	157 (49.5)	73 (51.8)	
Race, Indian (n, %)	75 (23.7)	33 (23.4)	
RAAS use (n, %)	30 (9.4)	22 (15.6)	0.057
Smoker (n, %)	16 (5.0)	14 (9.9)	0.683
Serum uric acid (umol/L)	301 ± 84	325 ± 87	0.011
HbA1c (%)	7.4 ± 1.7	7.5 ± 1.5	0.770
Baseline eGFR ( ml/min/1.73 m^2^)	93 ± 26	74 ± 15	<0.001
Weight, Kg	70 ± 12	68 ±13	0.063
Systolic BP, mmHg	144 ± 18	146 ± 18	0.204
Diastolic BP, mmHg	88 ± 10	87 ± 10	0.210
Achieved BP* control, (n, %)	55 (17.3)	15 (10.6)	0.050
Diabetics hypertensive, (n, %)	89 (28.0)	58 (41.1)	0.004

BP: blood pressure, eGFR: estimated glomerular filtration rate, RAAS: renin-angiotensin aldosterone system, *Target BP control is < 130/80 mmHg for Diabetes and < 140/90 mmHg for all others.

A multivariate logistic regression analysis was used to examine the factors associated with new onset CKD in hypertensives at the end of the 10-year period. Variables that were significant (p < 0.05) in the univariate analyses with CKD were entered into the model. Table 3 shows that being older, having lower baseline eGFR, higher serum uric acid level and the presence of diabetes amongst hypertensive patients were positively and significantly associated with the development of CKD. Hypertensive patients with concomitant diabetes were at 2.6 time odds more likely to develop CKD than hypertensive without diabetes.

**Table 3:** Predictors of new onset of chronic kidney disease at UMMC (n = 460)

**Independent variable**	Adjusted OR (95% CI)*	**p-value**
Baseline eGFR (per 1 mL/min decrease)	1.041 ( 0.943-0.979)	<0.001
Age (per 1 year increase)	1.123 (1.078-1.169)	<0.001
Presence of DM in HPT	2.621 (1.490-4.608)	0.001
Uric acid (per 1 umol/L increase)	1.004 (1.001-1.007)	0.018

OR: Odds Ratio, CI: Confidence Interval DM: diabetes, HPT: hypertension

* Adjusted for age, gender, serum uric acid, baseline estimated glomerular filtration rate and diabetes status in hypertension.

## Discussion

The decline rate in glomerular filtration in this cohort of hypertensive patients was −1.26 ± 2.4 ml/min/1.73 m^2^ per year. This is a surprise finding as previous studies have shown higher decline rates of −1.5 mL/ min per 1.73 m^2^ per year [13] in the hypertensive population. Our study, actually shows the decline rate in hypertensive patients to be almost the same as that of a normal population. One possible reason for our finding is the better BP achieved at the end of 10 years. There was a lowering of SBP of 10 mmHg from baseline. Furthermore, there was a significant improvement in BP controlled to target, from 15.2% in 1998 to 41.1% at the end of 10-years. The average numbers of antihypertensive agents also increased from a mean of 1.2 to 2.

Nearly a third developed new onset CKD at the end of the 10-year follow-up. This conversion rate is high [24]. Although the decline in eGFR between the fifth and tenth year was very slow the number who developed new onset CKD in this time period increased quite substantially from 18.9% to 30.1%. This was because a fairly large proportion of the patients (n = 57, 12.5%) were on the borderline of Stage 2 CKD and so were “converted” to CKD Stage 3 (eGFR <60 ml/min per 1.73 m^2^) even with 1 ml/min decline between 2002 and 2007. None of the patients developed ESRD or needed renal dialysis. However, we still need to take cognizance of this high conversion rate as we would need to make adjustments to medications that are used. Furthermore, we need to intensify our management to reach target goals so as to delay the new onset of CKD. It should also prompt us to use more ACEI or ARB if not already used, as these agents have been shown to slow down the progression to CKD [25,26]. Our study has also shown that one of the factors associated with the development of new onset CKD is the presence of diabetes in hypertension. Many studies have reported similar findings [13,27]. One of the reasons for an increased deterioration in glomerular filtration is that diabetes itself causes renal damage, and the added presence of hypertension amplifies the vascular damage leading to further renal insufficiency [28-30].

Decline in eGFR have been reported to be higher in patients with untreated hypertension and diabetes ranging from −10 to −15 ml/min/year [31-34]. The rate of decline has also been shown to be slowed to between −2 to −5 ml/min/year with intensive antihypertensive therapy [32-35]. Our finding also showed diabetes at baseline was associated with an additional decline of −0.72 ml/min/year, resulting in an overall decline of −1.98 ml/min/year. This is much lower than that reported in other studies [32-35]. This is could be due to the lower BP values achieved in our study.

Our study indicated that baseline uric acid level is a predictor for the progression of renal impairment. There is an increasing body of evidence as well as controversies regarding the role of uric acid in progression of CKD [36-38]. One possible explanation that hyperuricaemia causes CKD is because of uric acid nephropathy itself [39]. A previous study has also shown that lowering serum uric acid level with allopurinol slowed down the progression of renal disease [40,41]. However, in another study, serum uric acid was not an independent predictor for CKD progression [37]. The weak positive association seen in our study could be due to our larger sample size (460 versus 177 subjects) and longer duration (10-year versus 7-years) compared to the other study [37].

Our findings of older age and lower baseline eGFR being the predictors of new onset CKD reaffirmed similar findings in previous studies, which showed that renal function declines as we age.

The initial BP level showed no correlation with new onset CKD in our study. This could be due to the baseline SBP being already quite low at 145 mmHg and being progressively lowered further over the 10-year period (Table 1) to 142 mmHg at 5 years and 135 mmHg at 10 years. Furthermore the BP control rate improved from 15.2% at baseline to 18.9% at 5 years and 41.1% at 10 years. This is consistent with another study which also failed to show significant relationship between BP and the development of CKD [25]. Furthermore the ONTARGET study showed that while combination therapy of ACEI and ARB had a greater BP reduction, they also significantly increased the incidence of renal dysfunction and hyperkalemia [42].

Studies have shown that the decline in glomerular filtration or the development of end stage renal disease was delayed with the use of drugs acting on the of renin-angiotensin system (RAS) like ACEI or ARB [25,26]. However, our study failed to show this. This is probably because of the low usage of RAS blockers at the beginning of the study (11.3%) and even at the end of 10 years where less than half were on RAS blockers. Although the use of RAS blockers increased over time, no one was on a combination of ACEI and ARB. Furthermore RAS was introduced rather late and at a more advanced stage of CKD. This was because of limited access to these agents in our centre.

### Strength and limitations

Our present study has several strengths and some limitations. Few studies have looked at changes in GFR in treated hypertension over a long of period of time. Our study provides further information on the progression of kidney function in hypertension, particularly in a primary care setting, where the majority of hypertensive patients is managed and hence will reflect more closely daily clinical practice. Our findings will also help to prognosticate the rate of decline in renal function in treated hypertension, and hence help us to optimize management in hypertensive patients in order to reduce cardiovascular events.

Our study has several limitations. Firstly, this was a retrospective study; therefore, not all relevant data was available. For example, screening for albuminuria was not done in many of the patients and hence this was not included in our analysis. While it is acknowledged that the presence of proteinuria is one of the strongest predictors of CKD and end stage renal failure [43,44], a metanalysis has shown that regressing proteinuria will not necessarily reduce the actual number of cardiovascular events [45]. Another limitation is not all patients had the complete 10-year follow-up and, hence some bias in the analysis may have been introduced. However, we do not think this will affect our findings in any substantial manner as the clinical characteristics of patients who did not have a complete 10 year follow-up or patients who were excluded due to missing data, were similar to those of who had complete data and the 10-year follow-up.

We evaluated renal function by using eGFR formula and not the gold standard of inulin test. Inulin test is expensive, not practical and will be out of the scope of daily clinical practice. We chose the Cockcroft-Gault formula over other commonly used formulae like the MDRD because the majority of our patients had higher eGFR and it is recognised that the MDRD formula is less accurate at higher levels of eGFR [46].

## Conclusions

The rate of decline in glomerular filtration in treated hypertensive patients is comparable to normal subjects. However, the likelihood of patients developing new Stage 3 or beyond CKD over a 10-year period is high. A lower baseline eGFR, hyperuricaemia, presence of diabetes and older age are independent risk factors for the development of CKD among patients with hypertension. These results suggest the need to detect new onset CKD in hypertensive patients, so that appropriate therapeutic strategies can be in place in order to reduce CVD morbidity and mortality in these groups of patients.

## Competing interests

The authors declare that they have no competing interests.

## Authors’ contributions

CYC contributed to the conceptualizing of the paper, data entry and writing of the manuscript while CSM contributed to data analysis and writing of the manuscript. CYC is the corresponding author. Both the authors read and approved the final manuscript.

## Pre-publication history

The pre-publication history for this paper can be accessed here:

http://www.biomedcentral.com/1471-2369/13/173/prepub
